# Tumor Decelerating and Chemo-Potentiating Action of Methyl Jasmonate on a T Cell Lymphoma *In Vivo*: Role of Altered Regulation of Metabolism, Cell Survival, Drug Resistance, and Intratumoral Blood Flow

**DOI:** 10.3389/fonc.2021.619351

**Published:** 2021-02-25

**Authors:** Yugal Goel, Saveg Yadav, Shrish Kumar Pandey, Mithlesh Kumar Temre, Babu Nandan Maurya, Ashish Verma, Ajay Kumar, Sukh Mahendra Singh

**Affiliations:** ^1^ School of Biotechnology, Institute of Science, Banaras Hindu University, Varanasi, India; ^2^ Department of Radiodiagnosis and Imaging, Institute of Medical Sciences, Banaras Hindu University, Varanasi, India; ^3^ Department of Zoology, Institute of Science, Banaras Hindu University, Varanasi, India

**Keywords:** methyl jasmonate, tumor growth impedance, cell survival and metabolic regulation, chemo-potentiation, intra-tumoral blood flow

## Abstract

Methyl jasmonate (MJ), a natural oxylipin, possesses a broad spectrum of antineoplastic potential *in vitro*. However, its tumor growth impeding and chemo-potentiating action has not been adequately investigated *in vivo*. Using a murine thymus-derived tumor named Dalton’s Lymphoma (DL), in the present study, we examined if intra-tumoral administration of MJ can cause tumor growth impedance. We also explored the associated molecular mechanisms governing cell survival, carbohydrate & lipid metabolism, chemo-potentiation, and angiogenesis. MJ administration to tumor-transplanted mice caused deceleration of tumor growth accompanying prolonged survival of the tumor-bearing mice. MJ-dependent tumor growth retardation was associated with the declined blood supply in tumor milieu, cell cycle arrest, augmented induction of apoptosis and necrosis, deregulated glucose and lipid metabolism, enhanced membrane fragility of tumor cells, and altered cytokine repertoire in the tumor microenvironment. MJ administration modulated molecular network implicating Hsp70, Bcl-2, TERT, p53, Cyt *c*, BAX, GLUT-1, HK 2, LDH A, PDK-1, HIF-1α, ROS, MCT-1, FASN, ACSS2, SREBP1c, VEGF, cytokine repertoire, and MDR1, involved in the regulation of cell survival, carbohydrate and fatty acid metabolism, pH homeostasis, and drug resistance. Thus, the present study unveils novel molecular mechanisms of the tumor growth decelerating action of MJ. Besides, this preclinical study also establishes the adjunct therapeutic potential of MJ. Hence, the present investigation will help to design novel anti-cancer therapeutic regimens for the treatment of hematological malignancies.

## Introduction

Global research efforts are focused on devising novel anti-cancer therapeutic strategies capable of specifically targeting the neoplastic cells. Thus, several agents capable of targeting one or more cancer hallmarks are being examined for their therapeutic efficacy. Methyl jasmonate (MJ), a natural oxylipin (Methyl(1R,2R)-3-Oxo-2-(2Z)-2-pentenyl-cyclopentaneacetate; http://www.chemspider.com/Chemical-Structure.4445210.html), possesses a promising antineoplastic potential, devoid of any harmful effects on healthy cells. The broad-spectrum anti-cancer action of MJ has been vividly demonstrated against neoplastic cells of diverse origins (1, 2). The cytotoxic activity of MJ against neoplastic cells has been shown to implicate a wide variety of molecular mechanisms but majorly through the induction of mitochondrial-mediated apoptotic cell death by involving altered functions of mitochondria (3, 4) and causing detachment of hexokinase from VADC (5). However, the bulk of data associated with MJ’s antineoplastic action is mainly from *in vitro* studies performed against a wide variety of neoplastic cell lines (1, 2, 6, 7). However, to optimally realize its chemotherapeutic potential to establish MJ as a standard anti-cancer drug, it is essential to assess the *in vivo* anti-cancer therapeutic efficacy, which is mainly lacking in the case of MJ.

*In vivo* based investigations are crucial for understanding various critical issues related to a drug’s ability to decelerate tumor progression. Moreover, other decisive aspects regulating tumor growth like dose, administration route, the bioavailability, mechanism(s) of action, side effects, toxicity, and different *in vivo* interactions also need to be worked out. However, only sporadic studies are available in this respect for MJ. The *in vivo* antitumor action of MJ has been investigated against only a limited type of malignancies, which includes breast cancer (8, 9), multiple myeloma (10), and murine lymphoma (11). However, these studies do not provide detailed mechanisms of *in vivo* antitumor efficacy/tumor growth retarding and chemo-potentiating MJ action. Thus, it is essential to investigate MJ’s effect on the crucial cancer cell-specific metabolism & cell survival regulation, membrane stability, angiogenesis, and drug resistance in an appropriate *in vivo* model. This will help to achieve therapeutic optimization laying the foundation for further clinical trials. However, except for only one study mentioned above (1), to the best of our knowledge, so far, there has been no comprehensive investigation revealing mechanisms of the therapeutic potential of MJ against any progressive hematological neoplasms.

Additionally, it will also be essential to understand the efficacy of MJ’s combinatorial use with other conventional anti-cancer drugs, which is only feebly investigated (1, 9). However, all of these studies were only *in vitro* based, with little clue about such combinations’ therapeutic efficacy under *in vivo* tumor-bearing situation. Hence, it is also essential to work out the *in vivo* therapeutic potential of MJ in a combinatorial study and the associated mechanism(s), which will lower the doses and, subsequently, the massive side effects of standard anti-cancer drugs.

Considering the lack of knowledge regarding the *in vivo* antineoplastic and chemo-potentiation mechanisms of MJ, the present study was conducted on a thymus-derived murine tumor, Dalton’s lymphoma (DL), to unravel the unknown mechanistic pathways. DL has been successfully used in our and other laboratories for exploring the therapeutic efficacy of several anti-cancer agents (12, 13). This is the first report on understanding MJ’s therapeutic efficacy on a progressively growing thymoma, with a comprehensive investigation of the underlying unexplored molecular mechanisms and issues related to MJ’s chemo-potentiating action *in vivo*.

## Materials and Methods

### Tumor, Mice, and Reagents

Dalton’s lymphoma initially discovered in the laboratory of A.J Dalton (NCI, Bethesda, USA) as a spontaneously originated thymoma in mice (14) and subsequently adapted for serial transplantation and ascitic growth (15), was used in the present investigation to understand antitumor action of MJ. We maintained this tumor in our laboratory by serial intraperitoneal transplantation in BALB/c mice for achieving ascitic tumor growth. Healthy male mice (6–8 weeks of age) were intraperitoneally transplanted with 5 x 10^5^ cells. Mice fully grown ascitic tumor is apparent by 12–16 days following tumor transplantation. The average survival time of tumor-bearing mice is usually 22 ± 2 days under normal conditions. Mice handling and experimentations were carried out as per approval of the institutional animal ethical committee (Approval reference: BHU/DOZ/IAEC/2018-2019/019; dated: January 01, 2019). HuT-78 and J6 (human T cell lymphoma) cell lines were obtained from NCCS, Pune, India. Methyl Jasmonate was commercially procured from Sigma-Aldrich (USA) with 95% purity (catalog no. 392707). Most of the reagents were procured from Sigma-Aldrich (USA). Other reagents and biologicals were obtained from the following sources: Fetal calf serum (Hyclone, USA), and Annexin V/PI apoptosis detection kit (Imagenex, USA), Antibodies (Imagenex, USA, Sigma Aldrich, USA, Chemicon, USA, BD Pharmingen Inc, USA and e-Biosciences, USA), and Primers for RT-PCR (Eurofins, USA).

### Administration of MJ to Tumor Transplanted Mice

**Figure 1:**
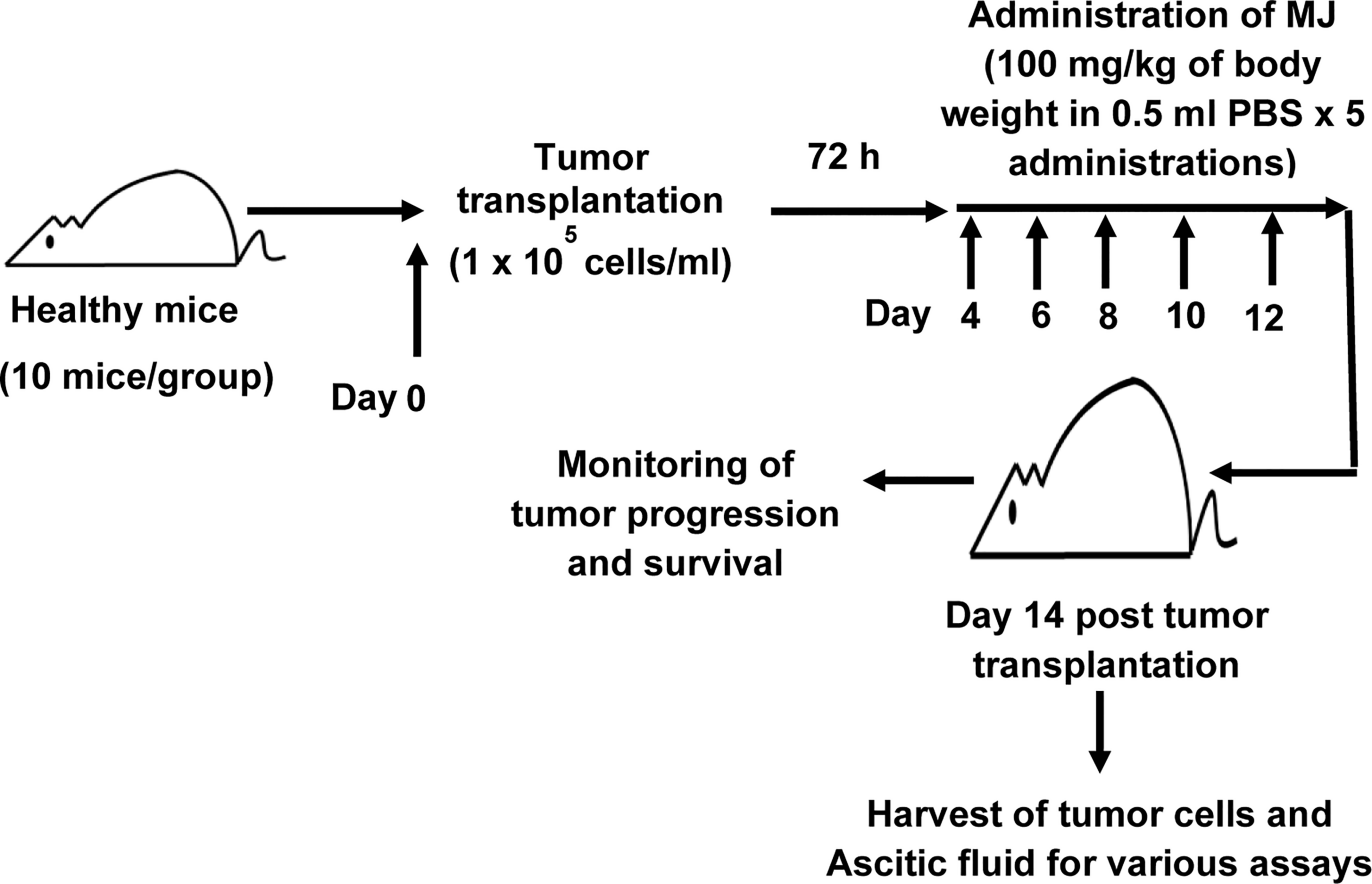
Protocol for the administration of MJ to tumor-bearing mice. Mice in groups of 10 each were transplanted with 1 x 10^5^ DL cells in 0.5 ml PBS per mouse (day 0), followed by intraperitoneal administration of MJ (100 mg/kg) or PBS on days 4, 6, 8, 10, and 12 post tumor transplantation. Tumor cells and ascitic fluid were collected and investigated for various assays on day 14. Remaining tumor-bearing mice were monitored for change of body weight and survival as parameters of tumor progression.

To study the effect of MJ administration on tumor progression, we followed a protocol summarised in **Figure 1**. Tumor cells were harvested from the tumor-bearing mice administered with PBS alone or containing MJ (100 g/kg) on the indicated days. The remaining mice were left to study the rate of tumor progression and survival time. As body weight is reported to be a parameter for tumor progression in ascitic tumor growth (16), the same was used in the study to monitor tumor progression along with an estimation of the total volume of ascitic fluid and enumeration of viable tumor count (13). In each group, 10 mice were used.

### Dye Exclusion Test for Cell Survival

The number of viable tumor cells was enumerated using the standard trypan blue dye exclusion test (17).

### MTT Assay

The MTT assay was used for estimating metabolic activity and calculated cytotoxicity following the method described by Mosmann (18), with slight modifications. The MTT solution (final concentration 0.5 mg/ml) was added to the wells containing the tumor cells in the medium, and further incubation was carried out for 4 h (37 °C in CO_2_ incubator). The formazan crystals formed during the incubation period were solubilized using DMSO for overnight. Absorbance at 540 nm was measured by the ELISA plate reader (Labsystems Finland). ID_50_ values were calculated by following a method described by Vertosick et al. (19).

### Estimation of Cytotoxicity

Cytotoxicity was assayed using a method described earlier (20), using MTT assay. The percentage of cytotoxicity was calculated using the following formula:

$\begin{array}{l} \text{\% Cytotoxicity}=\frac{A-B}{A}\times 100\\ A:\;Absorbance\;Control\\ B:\;Absorbance\;MJ \end{array}$

### Estimation of Cell Death

The apoptotic and necrotic modes of cell death were evaluated by microscopic observation of the control, and MJ exposed tumor cells using the standard Wright-Giemsa and Annexin V/PI staining (17, 21) following the manufacturer’s instructions.

#### Cell Cycle Analysis

The cell cycle analysis was performed following a method described earlier (17) based on the method of Shen et al. (22). The tumor cells harvested from the tumor-bearing mice of control and treated groups were washed twice with chilled PBS, fixed in 70% ice-cold ethanol followed by incubation for 30 min at −20°C. The cells (1×10^5^ cells/ml) were then washed with PBS, stained with PI (10 μg/ml) following incubation with 20 μg/ml of RNAase A solution for 30 min at 37°C in dark. Thereafter, cells were analyzed by the flow cytometer for cell cycle arrest. A total of 10,000 cells per sample was acquired on a flow cytometer (BD FACSCalibur) using a PI suitable band filter and the events below 400/Sec. The primary gate was on a dot plot with FSC vs. SSC. A secondary gate was applied around the FL2-A (pulse area) vs. FL2-W (pulse width). Finally, the data was recorded on a histogram plot with FL2-A at X-axis & cell counts at Y-axis, and analysis was quantified by the Cell Quest software (Becton Dickinson).

### Detection of Cell Surface Expression of MCT-1, GLUT-1, and MDR1

The expression of MCT1, GLUT-1, and MDR1 was analyzed by flow cytometry (23, 24) along with Western blotting (25). Cells were incubated with primary antibody for 40 min, washed, and subsequently incubated with fluorochrome-conjugated secondary antibody for 30 min at 37 °C in a humidified atmosphere. The expression pattern of the indicated molecules was analyzed by flow cytometry.

### Immunodetection of Cytokine Production in Ascitic Fluid

The levels of IL-6, IFN-Ƴ, and TGF-β were detected by a standard ELISA in the ascitic fluid of control and MJ-exposed group (26). The ELISA plate wells were coated with 50 µl of ascitic fluid and kept at 4 °C overnight. The ELISA plates were extensively washed with PBS-Tween-20 (0.1% v/v), followed by blocking of unsaturated sited by BSA (1% w/v) solution in PBS. Washing was followed by incubation with the primary antibody and then with alkaline phosphatase-conjugated secondary antibody at room temperature. After washing, p-nitrophenyl phosphate (1 mg/ml) was added, followed by incubation for 30 min at room temperature. Absorbance was measured at 405 nm.

### Immunodetection of Cytosolic Cyt *c*

Cytosolic Cyt *c* was immunodetected by Western blotting using cytosolic extract (27) as described earlier. Cell lysis was carried out by incubating cells in lysis buffer pH 7.5 [(HEPES 200 mM, MgCl_2_ 1.0 mM, sucrose 250 mM, EGTA 1.0 mM, PMSF 0.1 mM, DTT 1.0 mM, KCl 2 µg/ml, aprotinin and leupeptin (2 µg/ml)] followed by homogenisation. Lysate thus obtained was centrifuged (16,000 x g, 4° C, 20 min). The harvested supernatant was used for immunoblotting for the detection of Cyt *c*.

### Immunoblotting

Immunoblotting was carried out to detect the indicated proteins as described earlier (20, 25) using cytosolic extract, or cell lysates obtained by lysing cells for 30 min on ice with lysis buffer [(Tris-Cl 20 mM (pH 8.0), NaCl (137 mM), glycerol 10 (v/v), Triton X-100 1% (v/v), EDTA 2.0 mM, PMSF 1.0 mM, Leupeptin 20 mM, and aprotinin 0.15 U/ml)]. The standard Bradford method was used to estimate the protein content in cytosolic extract or cell lysates (28). Samples for loading of gel were prepared in a gel-loading buffer [(Tris-Cl 0.5 M (pH 6.8), β-mercaptoethanol 100 mM, SDS 20% (w/v), Bromophenol blue 0.1% (v/v), and glycerol 10% (v/v)] by heating on a boiling water bath for 3 min. Thirty µg protein per sample was loaded on the polyacrylamide-SDS gel for electrophoresis. After electrophoresis, transfer of proteins to the nitrocellulose membrane (Sartorius, Germany) was carried out, followed by incubation with primary antibodies against the respective proteins. After that, the membrane was incubated with an alkaline phosphatase-conjugated secondary antibody followed by BCIP/NBT solution to visualize bands. The intensity of each protein band was analyzed by ‘ImageJ’ software. β-actin was used as a loading control.

### Reverse Transcriptase-Polymerase Chain Reaction (RT-PCR)

The expression of indicated RNA was estimated using standard protocol for RT-PCR following a method described earlier (17). cDNA was prepared using a cell to cDNA kit as per the manufacturer’s instructions (Ambion, USA). Primers for the indicated genes were obtained from Eurofins; the detailed description is provided in **Table 1**. Thirty-five cycles of amplification were done with each comprising denaturation for 2 min at 94 °C, annealing at 55–60 °C as per the genes’ primers, and elongation for 30 s at 72 °C. The DNA bands were separated on 2% agarose gel containing ethidium bromide (0.25% w/v) by electrophoresis and then visualized using a UV-transilluminator. The band intensity of each gene was analyzed by “ImageJ” software. β-actin was used as a loading control.

**Table 1:** Primer sequence for RT-PCR.

Gene	Primer Sequence
**ACSS2**	F-5′GTGGATGAAAGGAGCAACTACA-3’; R-5′GCCCTCCCAGTAAAAAGCAACT-3’
**FASN**	F-5′-AGGGGTCGACCTGGTCCTCA-3’; R-5′-GCCATGCCCAGAGGGTGGTT-3’
**GLUT-1**	F-5’-CTTTGTGGCCTTCTTTGAAG-3’; R-5’-CCACACAGTTGCTCCACAT-3’
**LDH A**	F-5’-TGTCTCCAGCAAAGACTACTGT-3’; R-5’-GACTGTACTTGACAATGTTGGGA-3’
**PDK-1**	F-5′-CCGGGCCAGGTGGACTTC-3′; R-5′-GCAACTCTTGTCGCAGAAACATAA-3′
**HIF-1α**	F-5’-CTCAAAGTCGGACAGCCTCA-3’; R-5’-CCCTGCAGTAGGTTTCTGCT-3’
**β-Actin**	F-5’GGCACAGTGTGGGTGAC-3’; R-5’-CTGGCACCACACCTTCTAC-3’

### DCFDA Staining for Intracellular ROS Expression

Intracellular ROS expression was detected using DCDFDA staining as described by Furuta et al. with minor modifications (29). Tumor cells were incubated in HBSS containing DCFDA (0.1 mM) for 45 min at 37°C. After washing, DCFDA stained cells were observed under the fluorescence microscope (Nikon, Japan) at 400 X magnification. The fluorescence intensity was analyzed by “ImageJ”.

### Ultrasonographic Imaging

A transabdominal color Doppler was performed on control and MJ-administered mice for observing blood flow in the ascitic cavity with iU22 Ultrasound (Phillips, NL), as per the method described earlier (30). We used a linear multifrequency assay transducer (7–11 MHZ) for detecting alterations in blood flow in the peritoneal cavity (30).

### Statistical Analysis

Experiments were conducted thrice. The statistical significance of differences between test groups was analyzed by Student’s t-test. A p-value of less than 0.05 was considered as significant.

## Results

### MJ Administration Retards Tumor Progression

**Figure 2:**
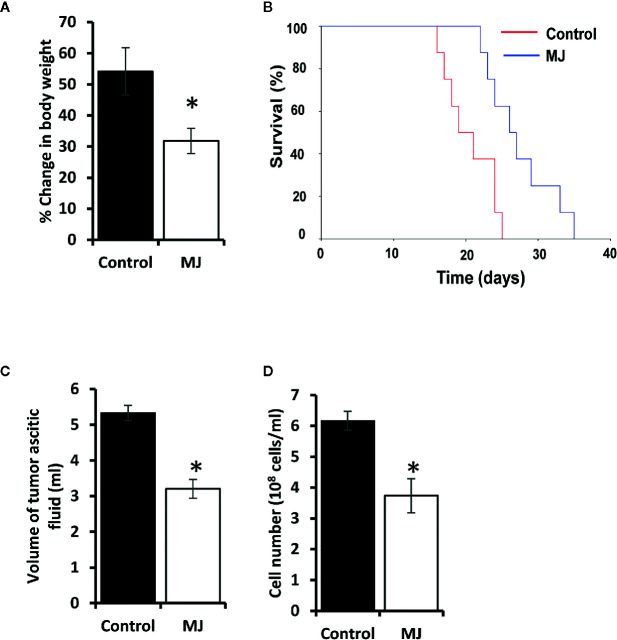
MJ administration retards tumor progression. Tumor-transplanted mice were administered with PBS alone (control) or containing MJ (100 g/kg), as shown in **Figure 1**, followed by monitoring of tumor progression by the change of body weight **(A)** and survival of the tumor-bearing mice **(B)**, estimation of the volume of ascitic fluid **(C)**, and the number of viable tumor cells **(D)**. Values are shown in **(A**, **C**, **D)** are mean ± SD of three independent experiments. *p < 0.05 vs. respective control.

Initial experiments were conducted to explore if MJ’s administration impedes tumor progression following the protocols as shown in **Figure 1**. Tumor growth was examined by estimating body weight and time duration of the host survival. Administration of MJ impeded tumor growth compared to the control (**Figure 2A**). The survival time of MJ-administered mice was also increased significantly compared to the untreated group (**Figure 2B**). To corroborate the observations mentioned above, the volume of ascitic fluid was measured as an indicator of tumor size. The number of tumor cells in the ascitic fluid was also counted. Administration of MJ caused a significant waning of tumor size (**Figure 2C**) compared to the control. Similarly, a substantial reduction in tumor cell numbers (number of tumor cells/ml of ascitic fluid) was also observed following MJ administration compared to control (**Figure 2D**). These two parameters were further confirmed the tumor growth decelerating action of MJ.

### MJ Displays Tumor Cell-Specific Cytotoxic Activity

**Figure 3:**
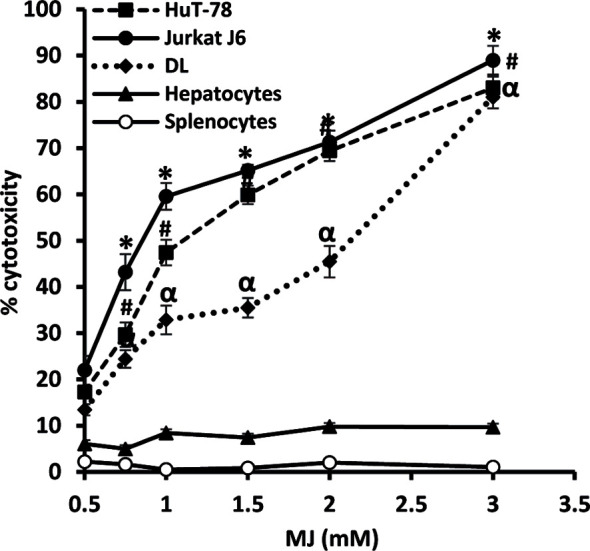
*In vitro* tumor cell-specific cytotoxic action of MJ against neoplastic cells of human origin. Indicated tumor cell lines, hepatocytes, and splenocytes (1 x 106 cells/ml) were incubated in vitro for 12 h in medium alone or containing MJ followed by estimation of cytotoxicity by MTT assay. Values shown are mean ± SD of three independent experiments.*,α, ^#^p < 0.05 vs. respective control.

To ascertain MJ’s tumoricidal action spectrum, we also examined its *in vitro* cytotoxicity against DL, HuT-78, and J6 cells, along with splenocytes and hepatocytes obtained from healthy mice. As shown in **Figure 3**, treatment of tumor cells with MJ resulted in a significant dose-dependent cytotoxic action against DL (ID_50_: 558 µg/ml), HuT-78 (ID_50_: 280 µg/ml), and J6 (ID_50_: 167 µg/ml) cells compared to their respective untreated controls. Further, *in vitro* exposure of normal cells, splenocytes, and hepatocytes to MJ, did not affect their survival (**Figure 3**).

### Molecular Mechanisms Associated With MJ-Dependent Tumor Growth Deceleration

**Figure 4:**
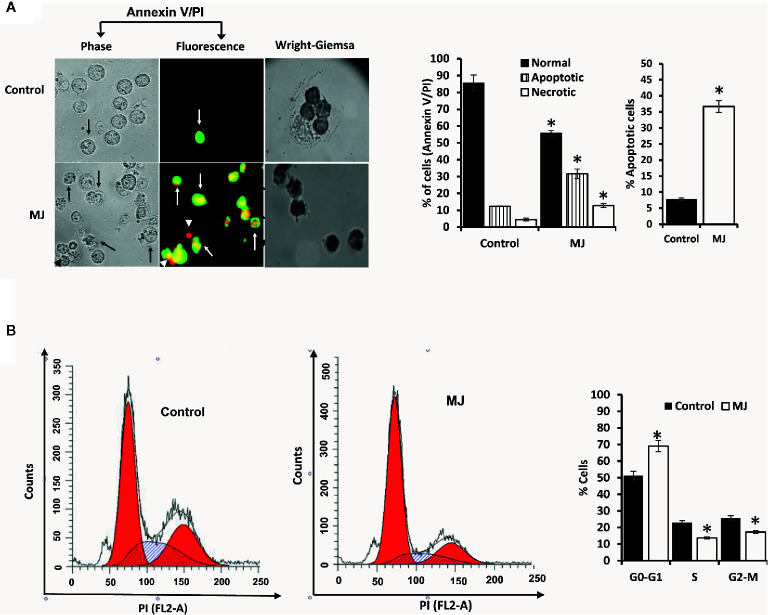
MJ administration to tumor-bearing mice induces tumor cell death and cell cycle arrest. Tumor cells harvested from control and tumor-transplanted mice were examined for induction of cell death by Annexin V/PI staining using fluorescence microscopy and Wright-Giemsa staining **(A)**. Arrows indicate apoptotic cells, and arrowheads indicate necrotic cells. Tumor cells were also analyzed for cell cycle as described in materials and methods using flow cytometry **(B)**. Microscopic and flow cytometric images are from a representative experiment of three independent experiments with similar results. The accompanying bar diagrams are mean ± SD three independent experiments. *p < 0.05 vs. respective control.

**Figure 5:**
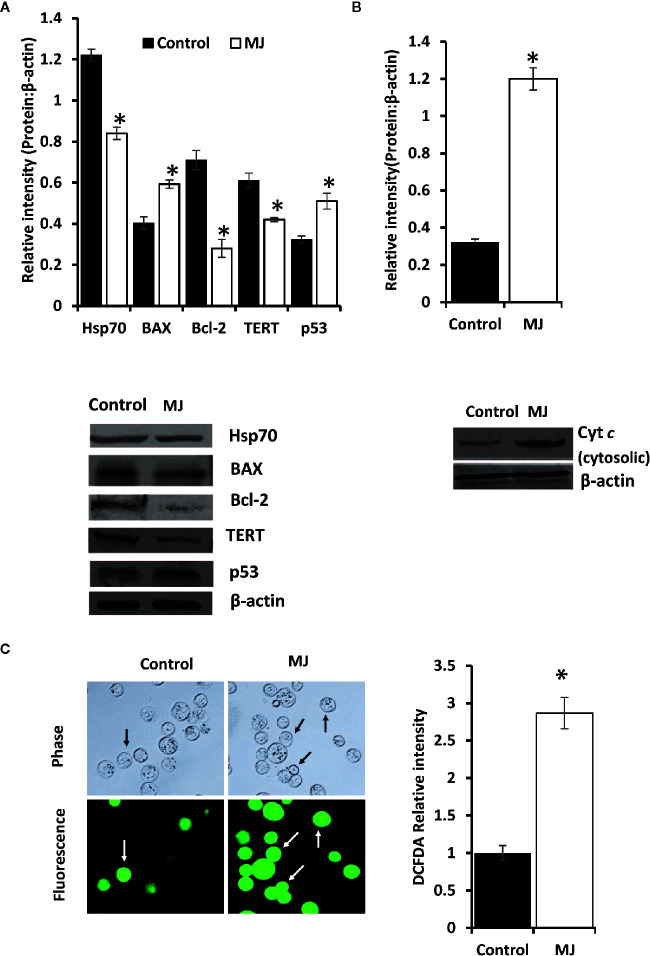
Altered expression of cell survival regulatory molecules in tumor cells following *in vivo* exposure to MJ administration. Tumor cells (1 x 10^6^ cells/ml) harvested from control and MJ-administered tumor-transplanted mice were analyzed for the expression of indicated cell survival regulatory molecules **(A)**. The cytosolic level of Cyt *c* was detected **(B)** as described in the materials and methods. Bands shown in **(A, B)** are from a representative experiment out of three independent experiments with similar results. Expression of intracellular ROS in tumor cells of control and MJ groups was estimated by DCFDA staining **(C)** as described in the materials and methods. Accompanying bar diagrams depicts densitometric analysis showing mean ± SD. *p < 0.05 vs. respective control.

One of the principal focus of this investigation was to decipher the molecular mechanisms underlying the observed tumor growth retarding action of MJ. We analyzed the mode of cell death induction, cell cycle, and expression repertoire of proteins that regulate these events. The number of apoptotic and necrotic cells showed a significant rise in the MJ group compared to the control (**Figure 4A**). These results indicate that MJ-administration triggers cell death *via* the induction of apoptosis and necrosis. As cell death is a direct consequence of cell cycle arrest (31), we also analyzed the cell cycle in tumor cells obtained from the control and MJ group of mice. Results presented in **Figure 4B** suggest that MJ arrests cell cycle in the G_0_/G_1_ phase. Because of these observations, next, we examined if the repertoire of proteins responsible for regulating cell cycle and apoptosis showed modulated expression in MJ-exposed tumor cells. Further, tumor cells harvested from the MJ group displayed a significantly declined Hsp70, Bcl-2, and TERT expression. In contrast, p53 and BAX expression levels were increased compared to tumor cells of control group (**Figure 5A**). Therefore, their modulated expression could underlie the observed altered survival of tumor cells exposed to MJ *in vivo*.

As altered mitochondrial membrane permeability is associated with the increased cytosolic release of Cyt *c*, reflecting the mitochondrial-dependent cell death induction, we also estimated the level of cytosolic Cyt *c* in MJ-exposed tumor cells. Results (**Figure 5B**) showed a significant increase in the level of cytosolic Cyt *c* in the MJ-exposed tumor cells compared to PBS-administered control (**Figure 5B**). As an increase of intracellular ROS is also implicated in the mitochondrial associated induction of apoptosis, next, we examined intracellular ROS level. We found dramatically increased intracellular ROS level in the tumor cells isolated from the MJ-administered group compared to the control group (**Figure 5C**).

### MJ Administration Manifests Inhibition of Glycolysis in Tumor Cells

**Figure 6:**
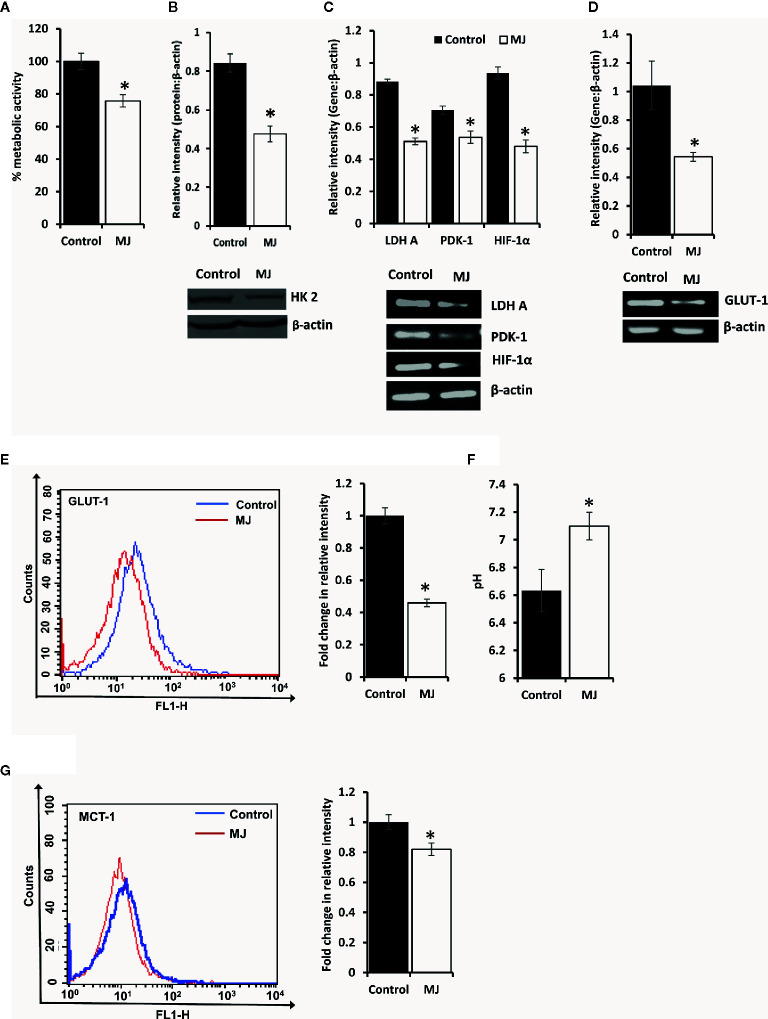
MJ alters the metabolic activity of DL cells *in vivo.* Tumor cells (1 x 10^6^ cells/ml) of control and MJ-administered tumor-bearing mice were analysed for metabolic activity by MTT assay **(A)**, expression of HK2 by Western blotting **(B)**, and other indicated metabolism regulatory molecules by RT-PCR **(C)**. The expression of GLUT-1 was analyzed by RT-PCR **(D)** and flow cytometry for membrane expression of GLUT-1 **(E)**. The pH of the control and MJ-administered groups’ ascitic tumor fluid was measured **(F)** by pH meter. The expression of pH regulator MCT-1 was analysed by flow cytometry **(G)** in tumor cells harvested from control and MJ-administered tumor-bearing mice. Bands shown **(B, C, D)** and flow cytometric image **(E, G)** are from representative experiments out of three independent experiments with similar results. Values shown in bar diagrams are mean ± SD of three independent experiments. *p < 0.05 vs. respective control.

To understand if MJ administration modulated tumor cells’ glucose metabolism as glycolysis has been noticed as one of the crucial regulators of apoptosis and necrotic cell death (3). Therefore, MJ administration’s effect on the metabolic activity (**Figure 6A**) and repertoire of glucose metabolism regulatory molecules (**Figures 6B–E**) were examined. The results showed significant inhibition of metabolic activity in tumor cells exposed to MJ compared to PBS administered control. The inhibition of metabolic activity was accompanied by a suppressed expression of glucose metabolism regulatory molecules, namely HIF-1α, HK 2, LDH A, PDK-1, and GLUT-1.

As carbohydrate metabolism is intimately associated with altered pH homeostasis in neoplastic cells, MJ administration’s effect on the pH of ascitic tumor fluid was also investigated. The MJ group’s ascitic fluid displayed relative alkalinization compared to the control group (**Figure 6F**). This suggests a reversal of the acidic tumor microenvironment. Considering the critical role of pH regulator MCT-1 in the maintenance of pH homeostasis in tumor cells through regulating the transport of lactate across the plasma membrane, next, we checked the expression of MCT-1. The MJ-exposed group’s tumor cells showed inhibited MCT-1 expression compared to control group tumor cells (**Figure 6G**).

### MJ Exposure Alters Lipid Metabolism in Tumor Cells

**Figure 7:**
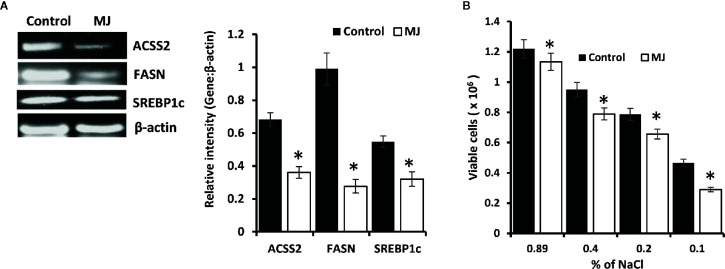
MJ alters lipid homeostasis. Tumor cells (1 x 10^6^ cells/ml) harvested from control and MJ administered tumor-bearing mice were analysed for the expression of ACSS2, FASN, and SREBP1c by RT-PCR **(A)**. Osmotic fragility **(B)** of control and MJ exposed tumor cells *in vivo* was analyzed, as described in the materials and methods. Bands shown in **(A)** are from a representative experiment out of three independent experiments with similar results. Values shown in bar diagrams **(A, B)** are mean ± SD of three independent experiments. *p < 0.05 vs. respective control.

Because lipid homeostasis is also impacted by altered carbohydrate metabolism and lipid biogenesis is a lifeline of tumor cell proliferation (20). Therefore, the effect of MJ on lipid metabolism was evaluated. Thus, the expression of lipid metabolism-regulating molecules was analyzed (**Figure 7A**). Significant down-regulated expression of fatty acid synthase (FASN) was observed in the MJ group compared to control, a key regulatory enzyme of *de novo* fatty acid synthesis (**Figure 7A**). Similarly, MJ-exposed tumor cells displayed inhibited expression of ACSS2, and SREBP1c (**Figure 7A**). The impact of inhibited expression of FASN, ACSS2, and SREBP1c in MJ-exposed tumor cells was also investigated by assessing tumor cell membrane’s stability through examining the osmotic fragility (**Figure 7B**). MJ-exposed tumor cells showed a significant increase in osmotic fragility compared to control.

### Altered Cytokines Repertoire in Ascitic Fluid

**Figure 8:**
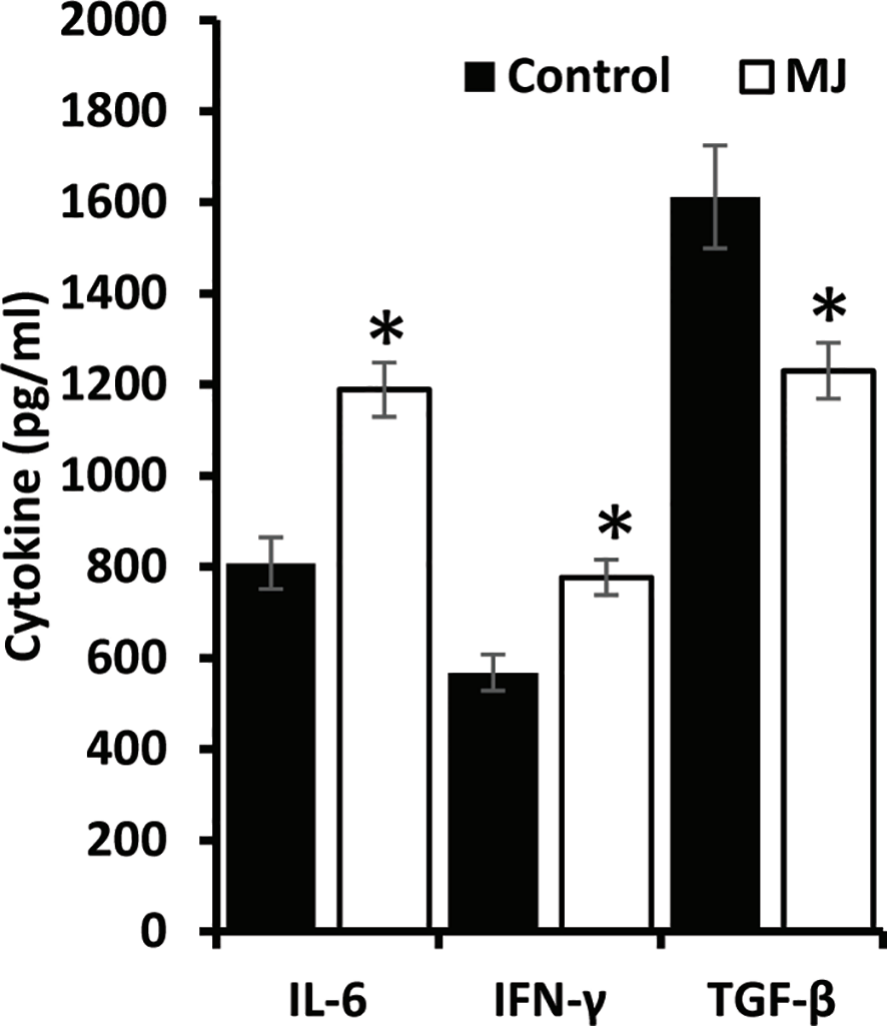
MJ administration to tumor-bearing mice alters cytokine repertoire in ascitic fluid. The ascitic fluid of control and MJ administered tumor-bearing mice were analyzed for the levels of indicated cytokines by ELISA as described in the materials and methods. Values are mean ± S.D. *p < 0.05 vs. control.

ELISA was performed on the ascitic fluid obtained from control and MJ groups to immunodetect the indicated cytokines (**Figure 8**). The MJ group’s ascitic fluid showed elevated IL-6 and IFN-Ƴ, accompanied by declined TGF-β levels compared to the PBS-administered control.

### MJ Administration Modulates VEGF Expression and Intra-Tumoral Blood Flow

**Figure 9:**
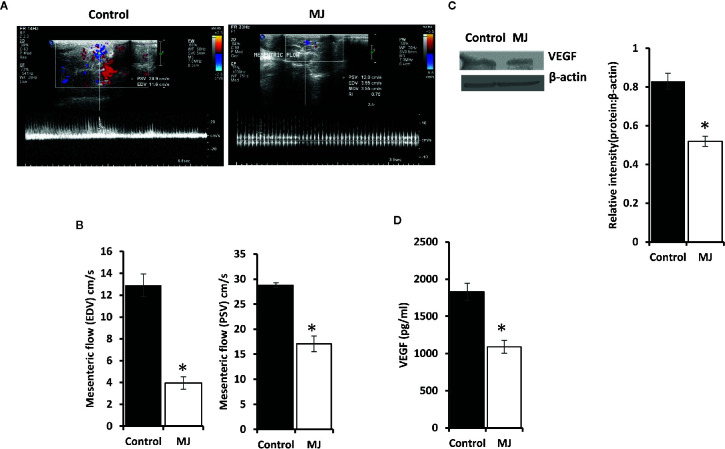
Effect of MJ administration on intratumoral blood flow and VEGF expression. A color doppler sonographic imaging was performed on control and MJ-administered tumor-bearing mice, as described in materials and methods. Images of Peak Systolic Velocity (PSV) and End Diastolic velocity (EDV) shown in **(A)** are from a representative experiment out of three independent experiments with similar results. The accompanying bar diagrams **(B)** show the mean ± SD of PSV and EDV, respectively. Tumor cells (1 x 106 cells/ml) harvested from control and MJ-administered tumor-bearing mice were analysed for the expression of VEGF by Western blotting in cell lysate **(C)** and by ELISA in ascitic fluid **(D)**. Bands shown in **(B)** are from representative experiments out of three independent experiments with similar results. Values shown in bar diagrams **(B–D)** are mean ± SD of three independent experiments. *p < 0.05 vs. respective control.

A color doppler ultrasonographic imaging was performed to understand if MJ-dependant tumor growth retardation also changed the pattern of blood flow in the vicinity of progressively growing ascitic tumors in the peritoneal cavity. The mesenteric blood flow was quantified for the peak-systolic velocity (PSV) and end-diastolic velocity (EDV), which significantly declined after MJ-administration compared to control (**Figures 9A, B**). These observations indicate that MJ inhibits blood flow in the ascitic cavity. Considering VEGF’s implication in the regulation of tumor angiogenesis, we also examined if MJ could modulate VEGF level. Notably, the level of VEGF was found to decrease in MJ-exposed tumor cells (**Figure 9C**) and the ascitic fluid (**Figure 9D**) of the MJ group.

### Chemo-Potentiating Action of MJ

**Figure 10:**
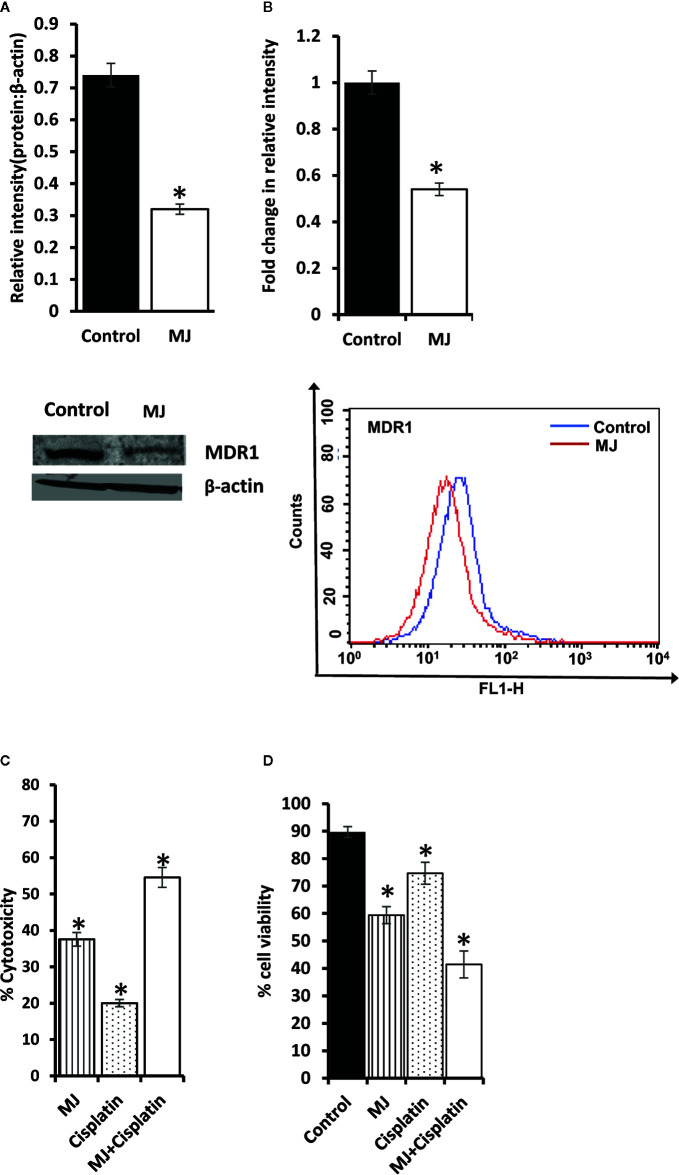
Chemo-potentiating action of MJ *in vivo*. Tumor cells (1 x 10^6^ cells/ml) harvested from control and MJ-administered tumor-bearing mice were examined for the expression of MDR1 by Western blotting **(A)** and flow cytometry **(B)**. Bands shown **(A)** and flow cytometric images **(B)** are from representative experiments out of three independent experiments with similar results. Values shown in bar diagrams are mean ± SD of three independent experiments. *p < 0.05 vs. respective control. To evaluate the chemo-potentiating effect of MJ administration, the tumor-bearing mice were administered with PBS with or without MJ (100 mg/kg body weight) or CP (5 mg/kg bodyweight) or both together on alternative days starting from day 4 to day 12 post tumor transplantation. Tumor cells harvested on day 14 from tumor-bearing mice of control and those administered with MJ, CP, and MJ plus CP were further examined for cytotoxicity by MTT assay **(C)** and survival by Trypan blue dye exclusion test **(D)** as described in materials and methods. Values shown are mean ± SD. *p < 0.05 vs. values for control.

As MJ on its own is not yet established as the drug of choice in the mainstream anti-cancer regimens, we checked if it can influence the tumor cell killing ability of standard anti-cancer drug cisplatin (CP) upon *in vivo* administration. Cell lysates were examined for MDR1 level by Western blotting (**Figure 10A**) and flow cytometry (**Figure 10B**). MJ-exposed tumor cells showed down-regulated expression of MDR1 compared to control. These observations indicate that *in vivo* exposure to MJ could enhance the cytotoxic ability of other anti-cancer drugs. This hypothesis was further confirmed by examining cytotoxicity (**Figure 10C**) and tumor cell viability (**Figure 10D**) in tumor cells isolated from tumor-bearing mice administered with PBS alone or containing MJ, CP, or a combination of both. Administration of MJ and CP markedly augmented cytotoxicity with a decreased number of viable cells compared to groups of cisplatin or MJ alone, indicating the chemo-potentiating effect of MJ for cisplatin.

## Discussion

Observations of the present study strongly suggest the tumor growth retarding action of MJ against a progressively growing lymphoma of thymic origin. Further, this investigation also explores the possible molecular mechanisms underlying the tumor growth impeding effect of MJ. Tumor cells harvested from MJ-administered tumor-transplanted mice displayed augmented induction of apoptosis and cell cycle arrest, which possibly led to a decline in tumor load. Further, *in vitro* exposure of MJ exhibited cytotoxic action against DL, HuT-78, and J6 cells without imparting any adverse effect on normal cells. This indicates the broad spectrum of tumor cell-specific cytotoxic action of MJ. Similar to our findings, other studies have shown that MJ does not harm normal cells (1, 32). Although the precise mechanism of the cell cycle interrupting and cell death-inducing action of MJ upon *in vivo* administration is not clear, one of the likely causes could be the altered expression of tumor metabolism regulatory molecules. MJ-exposed tumor cells showed inhibition of HK 2, LDH A, PDK-1, and HIF-1α expression, which have a pivotal role in maintaining the predominant glycolytic phenotype of tumor cells, crucially required for augmented survival of neoplastic cells (33–36). Studies have overwhelmingly demonstrated that MJ could bind to and detach mitochondria linked HK 2 in malignant cells (5), leading to the decoupling of glycolysis and mitochondrial metabolism (37), with detrimental consequences on cell survival.

MJ dependent dissociation of HK 2 from voltage-dependent anion channel (VDAC) is also reported to trigger a dip in mitochondrial transmembrane potential, leading to increase release of Cyt *c* and induction of apoptosis (37). Indeed, we also observed an augmented level of cytosolic Cyt *c* in tumor cells exposed to MJ *in vivo*, reconfirming the mitochondrial mode of cell death. The necrotic cell population’s surge also indicates the possibility of declined bioenergetics in MJ-exposed tumor cells, as induction of apoptosis is an ATP-dependent phenomenon (32). Thus, once ATP production is depleted in cells undergoing apoptosis, the necrotic mode of cell death can be ushered during the late stages of exposure to a metabolic inhibitor (17). The same could also underlie the inhibited expression of various metabolic molecules in MJ exposed tumor cells owing to increased cell death. Other studies have also indicated that hexokinase inhibited expression augments apoptosis induction, and declines cell proliferation (13, 37). Blocked ATP synthesis in MJ-treated hepatocellular carcinoma cells owing to inhibited metabolism has been demonstrated to cause necrosis in the neoplastic cells (3). Further, G_0_/G_1_ arrest of the cell cycle is associated with mitochondrial mode of apoptosis (38). MJ-dependent cell cycle arrest has also been demonstrated in other neoplastic cell lines exposed to MJ *in vitro* in the G_0_/G_1_ and S phase of the cell cycle. The difference in the phases of cell cycle arrest by MJ may depend on the type of tumor cells’ etiology (1).

We also observed an increase in intracellular ROS expression in tumor cells exposed to MJ *in vivo.* The augmented ROS level has been shown to induce apoptosis by causing the destruction of various macromolecules like proteins, lipids, and nucleic acids (39). Mitochondrial ROS triggers apoptosis induction *via* the intrinsic pathway, leading to activation of caspases (39). The increase of cytosolic Cyt *c* is reported to cause caspase activation (40). Caspase can also cause loss of mitochondrial functions and trigger an increased ROS generation (41).

There was no report regarding the effect of MJ *in vivo* on the expression of HIF-1α. HIF-1α plays a vital role in the reprogrammed cancer cell metabolism by enhancing the expression of GLUT-1, HK 2, LDH A, and PDK-1 (35). Hence, HIF-1α is considered both upstream and downstream master regulator of tumor metabolism (42). The inhibition of HIF-1α by exposure of tumor cells to MJ could induce mitochondrial passage of pyruvate, leading to activation of oxidative phosphorylation and ROS production with concomitant inhibition of GLUT-1, HK 2, LDH A, and PDK-1 axis (35), a lifeline of tumor metabolism. Nevertheless, LDH A inhibition causes a declined lactate production, leading to abrogation of tumor cell survival by disturbing the glycolytic bioenergetic homeostasis (33). HIF-1α also up-regulates the expression of pH regulator MCT-1 in tumor cells, regulating pH homeostasis (43, 44). Interestingly, we observed a reversal of tumor acidosis accompanied by a declined glucose uptake, glycolytic activity, and MCT-1 expression. As tumor acidosis is necessary to maintain cytosolic alkalinization of tumor cells, interference with the same is also reported to cause the induction of tumor cell death (45).

This is the first report to indicate the novel mechanism of *in vivo* antineoplastic action of MJ. We observed that MJ administration ushered modulation in the expression of crucial cell survival regulatory molecules, notably the declined Hsp70 and Bcl-2, and increased p53 and BAX levels. Indeed, increased level of Bcl-2 and declined p53 level have been implicated in antagonizing the induction of apoptosis in tumor cells (46). Further, Bcl-2 also regulates HIF-1α protein stabilization in neoplastic cells (47). Moreover, suppression of TERT leads to inhibition of cell proliferation and promotion of cell cycle arrest and apoptosis induction (48). Furthermore, TERT is reported in apoptosis regulation *via* Bcl-2 and p53 (49, 50).

Another interesting finding of the present study is the lipid metabolism inhibitory ability of MJ. The reason behind examining the effect of MJ on lipid metabolism was due to its indispensable role in the cell proliferation. We observed the declined expression of lipid homeostasis regulating molecules like FASN, ACSS2, and SREBP1c in MJ exposed tumor cells. These molecules play a pivotal role in sustaining the *de novo* fatty acid production in neoplastic cells (51, 52). Nevertheless, HIF-1α and Hsp70 directly regulate apoptosis in neoplastic cells (33, 53) and govern the expression of FASN, ACSS 2, and SREBP1c (51, 54). The consequence of the inhibition of these crucial lipid homeostasis regulating molecules in MJ exposed tumor cells *in vivo* is reflected in increased osmotic fragility of tumor cells, indicating hampered membrane biogenesis (51). Indeed, Yousefi et al. reported that MJ increases breast cancer cell apoptosis by causing a decline in membrane fluidity (9). In turn, membrane fluidity is regulated by the membrane’s lipid constituents (55). The declined membrane fluidity caused by MJ can also be associated with the hampered cell cycle progression (55).

It is mention-worthy that in one of our previous studies, curcumin, a well-established phytochemical with promising cancer therapeutic potential, also caused retardation of DL progression, implicating a modulation of the tumor growth-regulating constituents of the tumor microenvironment and induction of cell death by modulating the expression pattern of apoptosis, cell survival and chemoresistance regulatory molecules (56) as observed in the present investigation using MJ. Studies have also shown the role of anti-glycolytic and lipogenic inhibitory activity of curcumin in tumoricidal action (57, 58). Further, curcumin can alter mitochondrial membrane potential and the neoplastic cells’ inflammatory state (59–61). Thus, in these respects, MJ seems to be similar to curcumin concerning some of its tumor progression inhibiting mechanisms. Therefore, to fully realize the therapeutic potential of MJ, it will be beneficial to study the cooperation of MJ and curcumin in retarding tumor progression in appropriate *in vitro* and *in vivo* settings of DL and other cancer cells. Our preliminary unpublished and ongoing experimental observations indicate that MJ has an adjuvant effect in potentiating curcumin’s antitumor efficacy. Thus, there is a great potential to investigate the combined effect of these phytochemicals to optimize their successful application in anticancer regimens.

We also observed attenuated VEGF expression in tumor ascitic fluid and tumor cells of MJ-administered tumor-bearing mice. Indeed, VEGF is a well-defined target of HIF-1α (47). We also checked the pattern of blood flow in the ascitic tumor cavity using color doppler ultrasonographic imaging. Interestingly, we observed low mesenteric blood flow in MJ administered tumor-bearing mice. This observation indicates a declined blood flow in the tumor microenvironment, which could be responsible for tumor growth retardation. Moreover, diminished VEGF expression in tumor cells is also accompanied by augmented apoptosis (62). More so, inhibition of VEGF-dependent angiogenesis also induces tumor cell apoptosis (62). One report indicates the role of inhibited angiogenesis in MJ-induced cell death of endothelial and melanoma cells (63). However, the study used chick embryo angiogenesis as a model. The present study is the first of its kind to report the effect of *in vivo* administration of MJ on VEGF expression and blood flow in the tumor microenvironment of tumor-bearing mice. Further, we observed inhibition in the level of TGF-β in the ascitic fluid of MJ-administered tumor-transplanted mice, along with an increase in the level of IFN-Ƴ and IL-6. The inhibited expression of HIF-1α could also be associated with the increased expression of proinflammatory cytokines like IFN-Ƴ and IL-6 (17, 64). Both IFN-Ƴ and IL-6 promote antitumor immunity (13, 45) and have shown the inhibitory effect on the expression of HIF-1α (17, 20). However, the increased level of TGF-β is very well reported in the uninterrupted tumor cell progression through attenuation of tumor cell death and antitumor immune response (65).

We also observed the increased tumoricidal ability of cisplatin in the presence of MJ. Although there could be multiple factors underlying MJ’s action, we observed a declined expression of MDR1 in MJ exposed tumor cells. Indeed, MDR1 has been reported to manifest drug resistance to cisplatin (66). Moreover, HIF-1α regulates the expression of MDR1 (17, 67). Further, IFN-Ƴ has been shown to inhibit the expression of MDR1 (68). Numerous *in vitro* studies have shown the alteration in the expression of MDR1 by MJ (63). However, so far, no *in vivo* study had investigated the effect of MJ on MDR1 expression. The chemo-potentiating action of MJ has also not yet been evaluated in a progressively growing tumor. Hence, our study is the first to suggest MJ’s chemo-potentiating action against hematological origin’s malignancy and the plausible associated mechanisms.

**Figure 11:**
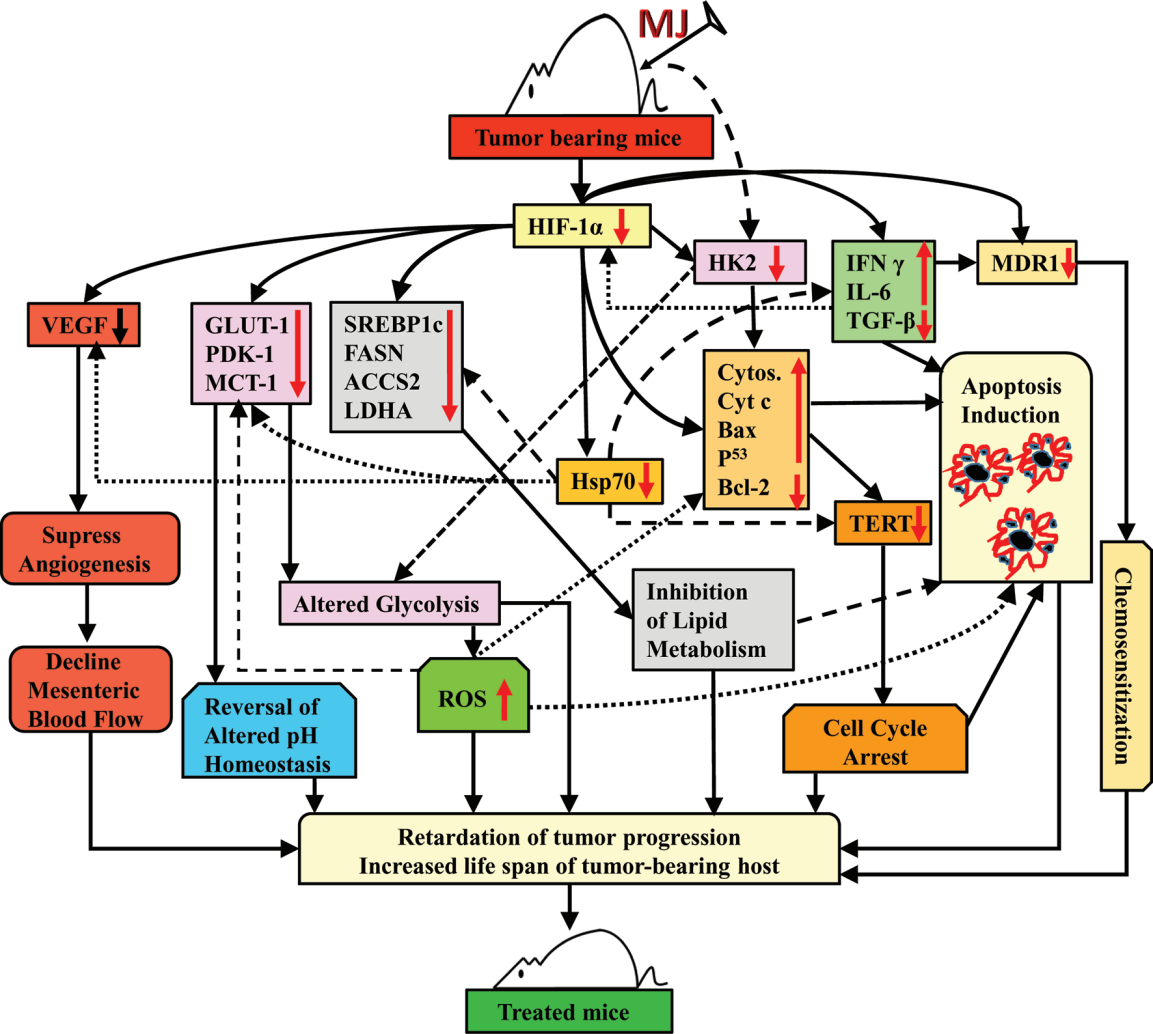
Summary of the molecular mechanism underlying the tumor growth retarding and chemo-potentiating action of MJ.

Taken together, the results of this study indicate that the administration of MJ to T cell lymphoma bearing mice retards tumor progression by triggering induction of tumor cell death associated with interference with carbohydrate and lipid metabolism, pH homeostasis, angiogenesis, and drug resistance. These actions of MJ are mainly re-laid on altered levels of HK2, HIF-1α, SREBP 1c, and MDR1 (**Figure 11**). This preclinical study’s findings will help to design the new anti-cancer therapeutic regimens against hematological origin malignancies.

## Data Availability Statement

The raw data supporting the conclusions of this article will be made available by the authors, without undue reservation.

## Ethics Statement

The animal study was reviewed and approved by the Institutional Animal Ethical Committee, Institute of Science, Banaras Hindu University, Varanasi 221005, UP, India (Approval reference: BHU/DOZ/IAEC/2018-2019/019 dated January 01, 2019).

## Author Contributions

YG: conceiving the idea, performing of experiments, data generation, interpreting data, and writing of the manuscript. SY: interpreting data and writing of the manuscript. SP: interpreting data and writing of the manuscript. MT: interpreting data and writing of the manuscript. BM: ultrasonography, interpreting data, and writing of the manuscript. AV: ultrasonography, interpreting data, and writing of the manuscript. AK: interpreting data and writing of the manuscript. SS: conceiving the idea, interpreting data, and writing of the manuscript. All authors contributed to the article and approved the submitted version.

## Conflict of Interest

The authors declare that the research was conducted in the absence of any commercial or financial relationships that could be construed as a potential conflict of interest.
